# Reconstruction of 3D knee MRI using deep learning and compressed sensing: a validation study on healthy volunteers

**DOI:** 10.1186/s41747-024-00446-0

**Published:** 2024-04-15

**Authors:** Thomas Dratsch, Charlotte Zäske, Florian Siedek, Philip Rauen, Nils Große Hokamp, Kristina Sonnabend, David Maintz, Grischa Bratke, Andra Iuga

**Affiliations:** 1 Department of Diagnostic and Interventional Radiology, Faculty of Medicine and University Hospital Cologne, University of Cologne, Kerpener Str. 62, Cologne, 50937 Germany; 2 Philips GmbH Market DACH, Röntgenstrasse 22, Hamburg, 22335 Germany

**Keywords:** Artifacts, Artificial intelligence, Deep learning, Knee joint, Magnetic resonance imaging

## Abstract

**Background:**

To investigate the potential of combining compressed sensing (CS) and artificial intelligence (AI), in particular deep learning (DL), for accelerating three-dimensional (3D) magnetic resonance imaging (MRI) sequences of the knee.

**Methods:**

Twenty healthy volunteers were examined using a 3-T scanner with a fat-saturated 3D proton density sequence with four different acceleration levels (10, 13, 15, and 17). All sequences were accelerated with CS and reconstructed using the conventional and a new DL-based algorithm (CS-AI). Subjective image quality was evaluated by two blinded readers using seven criteria on a 5-point-Likert-scale (overall impression, artifacts, delineation of the anterior cruciate ligament, posterior cruciate ligament, menisci, cartilage, and bone). Using mixed models, all CS-AI sequences were compared to the clinical standard (sense sequence with an acceleration factor of 2) and CS sequences with the same acceleration factor.

**Results:**

3D sequences reconstructed with CS-AI achieved significantly better values for subjective image quality compared to sequences reconstructed with CS with the same acceleration factor (*p* ≤ 0.001). The images reconstructed with CS-AI showed that tenfold acceleration may be feasible without significant loss of quality when compared to the reference sequence (*p* ≥ 0.999).

**Conclusions:**

For 3-T 3D-MRI of the knee, a DL-based algorithm allowed for additional acceleration of acquisition times compared to the conventional approach. This study, however, is limited by its small sample size and inclusion of only healthy volunteers, indicating the need for further research with a more diverse and larger sample.

**Trial registration:**

DRKS00024156.

**Relevance statement:**

Using a DL-based algorithm, 54% faster image acquisition (178 s *versus* 384 s) for 3D-sequences may be possible for 3-T MRI of the knee.

**Key points:**

• Combination of compressed sensing and DL improved image quality and allows for significant acceleration of 3D knee MRI.

• DL-based algorithm achieved better subjective image quality than conventional compressed sensing.

• For 3D knee MRI at 3 T, 54% faster image acquisition may be possible.

**Graphical Abstract:**

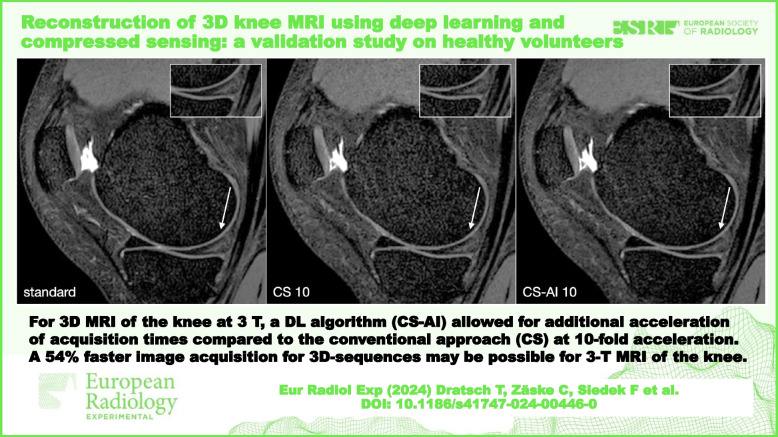

## Background

The knee is the second most common site of musculoskeletal pain [1]. Common knee injuries, such as anterior cruciate ligament (ACL) injuries, are highly prevalent in the general population [2, 3], leading to decreased productivity and diminished quality of life [4], while also imposing a substantial socioeconomic burden [5]. In this context, magnetic resonance imaging (MRI), with its ability to provide detailed tissue contrast, plays a critical role in the accurate diagnosis and evaluation of knee injuries [6]. However, the growing demand for MRI [7] has led to a bottleneck in terms of patient throughput, limiting the number of patients who can undergo timely examinations. To expedite image acquisition without compromising image quality, several innovative techniques have emerged. These include reduced scanning protocols [8], parallel imaging (*e.g.,* GRAPPA) [9], and compressed sensing (CS) [10]. Numerous studies have demonstrated the efficacy of CS in reducing scan times across various anatomical regions [11–14] while maintaining diagnostic image quality. However, conventional CS approaches face limitations in achieving higher acceleration factors due to the introduction of artifacts such as aliasing and blurring. Recent advancements in artificial intelligence (AI), especially deep learning (DL) have opened avenues for addressing these limitations by integrating it with conventional CS [15].

In the context of knee MRI, Pezotti et al. [15] introduced Adaptive-CS-Net as part of the 2019 fastMRI challenge which enabled an 8 × reduction in acquisition times. Adaptive-CS-Net is an AI-driven convolutional neural network that enhances MRI image reconstruction by integrating parallel imaging, CS, and DL, using iterative learning-based techniques to process undersampled k-space data, thereby improving image authenticity. Adaptive-CS-Net has since been further developed to accommodate a broader range of acceleration factors and anatomical regions, including the knee. In an initial study, Iuga et al. demonstrated a 64% reduction in scan time for two-dimensional knee MRI [16]. However, so far, there have been no studies testing the clinical feasibility and limitations of Adaptive-CS-Net to reconstruct three-dimensional (3D) MRI images of the knee. The implementation of this algorithm could help facilitate the routine clinical application of 3D-sequences, enabling enhanced visualization of anatomical structures not parallel to standard imaging planes, thereby improving diagnostic accuracy and efficiency. Thus, the aim of the current study was to compare the subjective image quality of MRI reconstructions using conventional CS and reconstructions using CS together with artificial intelligence (CS-AI) at different acceleration factors for 3D diagnostic MRI of the knee. The purpose was to determine whether CS-AI is a feasible approach to further decrease acquisition times of knee MRI while maintaining diagnostic image quality.

## Methods

### Study population

This prospective single center study was carried out in accordance with the ethical standards in the 1964 Declaration of Helsinki and its later amendments and was approved by our institutional review board. The study was registered in the national Clinical Trials Register (DRKS00024156). Recruitment of volunteers and acquisition of imaging data were carried out in March 2021. Written informed consent was obtained from all participants included in the study. Exclusion criteria were pregnancy, age below 18, implanted MRI conditional or unsafe devices, previous surgery or known pathologies of the knee, and knee related pain in the last 6 months.

### MRI acquisition and reconstruction

A whole-body 3.0-T MRI system (Philips Ingenia 3.0 T, Philips, Hamburg, Germany) with a dedicated knee coil (transmit/receive, 16 channels) was used for image acquisition. All volunteers were placed supine, feet-first on the table. For all sequences, the field-of-view covered the entire knee joint. The protocol included a fat saturated 3D proton density sequence with four different acceleration levels (10, 13, 15, and 17) as well as a sense sequence with an acceleration factor of 2. These specific levels were chosen to represent a broad spectrum of acceleration, from moderate to extremely high, allowing us to assess the efficacy of the CS-AI algorithm in maintaining image quality under varying degrees of data undersampling, corresponding to scan time reductions of 54%, 64%, 68%, and 73%, respectively. The selected acceleration levels were compared with the conventional sense sequence at an acceleration factor of 2, which is the standard 3D sequence used in our institution’s daily routine. Except for the acceleration factors, all other parameters were kept identical between the acquired sequences. Table 1 summarizes the sequence parameters used in this study, showing the scan time for each sequence and the potential reduction of scan time.

**Table 1:** Acquisition parameters for the different three-dimensional sequences and results for changes in the scan time

Sequence	**Standard** **SENSE**	**CS 10** **CS-AI 10**	**CS 13** **CS-AI 13**	**CS 15** **CS-AI 15**	**CS 17** **CS-AI 17**
Echo time [ms]	170	170	170	170	170
Repetition time [ms]	1,300	1,300	1,300	1,300	1,300
Flip angle [degrees]	90	90	90	90	90
Field of view [mm]	140 × 159 × 160	140 × 159 × 160	140 × 159 × 160	140 × 159 × 160	140 × 159 × 160
Slice thickness [mm]	3D sequence, therefore voxel size as volume				
Acquisition voxel size [mm]	0.63 × 0.62 × 0.63	0.63 × 0.62 × 0.63	0.63 × 0.62 × 0.63	0.63 × 0.62 × 0.63	0.63 × 0.62 × 0.63
Reconstruction voxel size [mm]	0.30 × 0.30 × 0.63	0.30 × 0.30 × 0.63	0.30 × 0.30 × 0.63	0.30 × 0.30 × 0.63	0.30 × 0.30 × 0.63
Turbo factor/echo train length	63	63	63	63	63
Sense/CS factor	2/ −	− /10	− /13	− /15	− /17
Scan time [s]	384	178	137	121	105
Saved scan time [s]		206	247	263	279
Scan time reduction [%]		54	64	68	73

Please note that only the acceleration factors were changed between the different sequences to keep them as comparable as possible. Also note that the acquisition times are the same for the CS and CS-AI sequences with the same acceleration factor. *CS* Acceleration using compressed sensing, *CS-AI* Acceleration using compressed sensing followed by AI image reconstruction using a deep learning-based algorithm

The sets of undersampled k-space data from the different acceleration levels were reconstructed into visually perceivable images using two methods: (1) a conventional approach with CS; and (2) a novel AI-driven prototype (CS-AI). The CS-AI-prototype is based on the “Adaptive-CS-Net” convolutional neural network, which employs an iterative, learning-based reconstruction scheme to process undersampled k-space data by exploiting additional information such as coil sensitivity and location of the image background, resulting in a combined algorithm of parallel imaging, CS and DL. Unlike the traditional wavelet transformation, the neural network performs sparsifying and consistency checks on the raw k-space data within each block, resulting in maximized image authenticity [17]. The algorithm presented by Pezzotti et al. [15] was extended by using training data of about 740,000 images with various anatomies, contrasts and field strengths (1.5 and 3 T). Both the acquisition and reconstruction algorithms of CS and CS-AI were provided by the manufacturer.

### Subjective image analysis

All scans were exported as Digital Imaging and Communications in Medicine − DICOM files to the clinical Picture Archiving and Communication System − PACS. Two board certified radiologists reviewed all images (R1, 8 years of experience; R2, 6 years of experience). For the subjective reading, the images were presented in random order and both readers were blinded to the scan sequence and reconstruction. All blinded images of a subject were available at once for both readers. Readers were free to choose window width and level settings and the review was performed over a period of 6 weeks.

Using a 5-point Likert scale, each reader independently evaluated delineation of the following anatomical structures for all sequences and reconstructions: anterior cruciate ligament, posterior cruciate ligament, menisci, cartilage and bone. Overall image impression and visible artifacts were rated additionally on a 5-point Likert scale, resulting in a total of seven subjective ratings for each of the nine images (CS 10/CS-AI 10, CS 13/CS-AI 13, CS 15/CS-AI 15, CS 17/CS-AI 17, and standard CS) reconstructed for every patient. Table 2 shows an overview of the used scale.

**Table 2:** Ratings for the anatomical structures, diagnostic certainty/overall image impression and artifacts used by the two readers for the evaluation of all sequences

Level	Anatomical structures	Overall image impression	Artifacts
1	Not visible/distinguishable	Not acceptable/no diagnostic value	Massive artifacts
2	Barely visible	Very limited diagnostic value	Significant artifacts
3	Adequately visible	Acceptable for most diagnoses	Acceptable artifacts
4	Good visibility	Good for majority of diagnoses	Minimal artifacts
5	Excellent visibility	Optimal	No artifacts

### Statistical analysis

GraphPad Prism version 9.0.1 for Mac OS X (GraphPad Software, Boston, United States) was used for all statistical analyses. For the subjective image analysis, the values from both readers for each sequence were averaged. To assess the interrater agreement between both readers, Krippendorff’s α was calculated, with a Krippendorff’s α ≥ 0.80 indicating high agreement, 0.67–0.79 indicating moderate agreement, and < 0.67 indicating poor agreement [18]. After assessing the normal distribution of the data using both graphical (*e.g.,* Q-Q plots and histograms) and statistical methods (D’Agostino and Pearson test [19]), robust mixed models fitted with the restricted maximum likelihood method − REML were employed to analyze the effect of acceleration level (10, 13, 15, and 17) and reconstruction method (CS *versus* CS-AI) on indicators of subjective image quality (overall impression, artifacts, and delineation of the anterior cruciate ligament, posterior cruciate ligament, menisci, cartilage and bone). As post hoc tests, Sidak’s multiple comparisons test was used to compare the different reconstruction methods (CS *versus* CS-AI) at the different acceleration levels. Additionally, Dunnett’s multiple comparisons test was used to compare all sequences to the reference sequence (standard sense with an acceleration level of 2). All post hoc tests were corrected for multiple comparisons. Data are reported as the mean ± standard deviation. A *p*-value below 0.05 was considered statistically significant. A priori sample size calculation was performed using G*power 3.1.9.7 based on previous results for acceleration techniques in knee imaging [12]. A minimum number of 19 volunteers were needed to detect a difference of 0.2 points on the Likert scale with 0.3 standard deviation, with an alpha error of 0.05 and a power of 0.80.

## Results

### Study population

Twenty young, healthy volunteers (12 males and 8 females; mean age 30.5 ± 2.1 years, range 24−43 years; mean weight 74 ± 12 kg, range: 55−100 kg; mean height 176 ± 11 cm, range 155−90 cm) were included.

### Image analysis

**Fig. 1:**
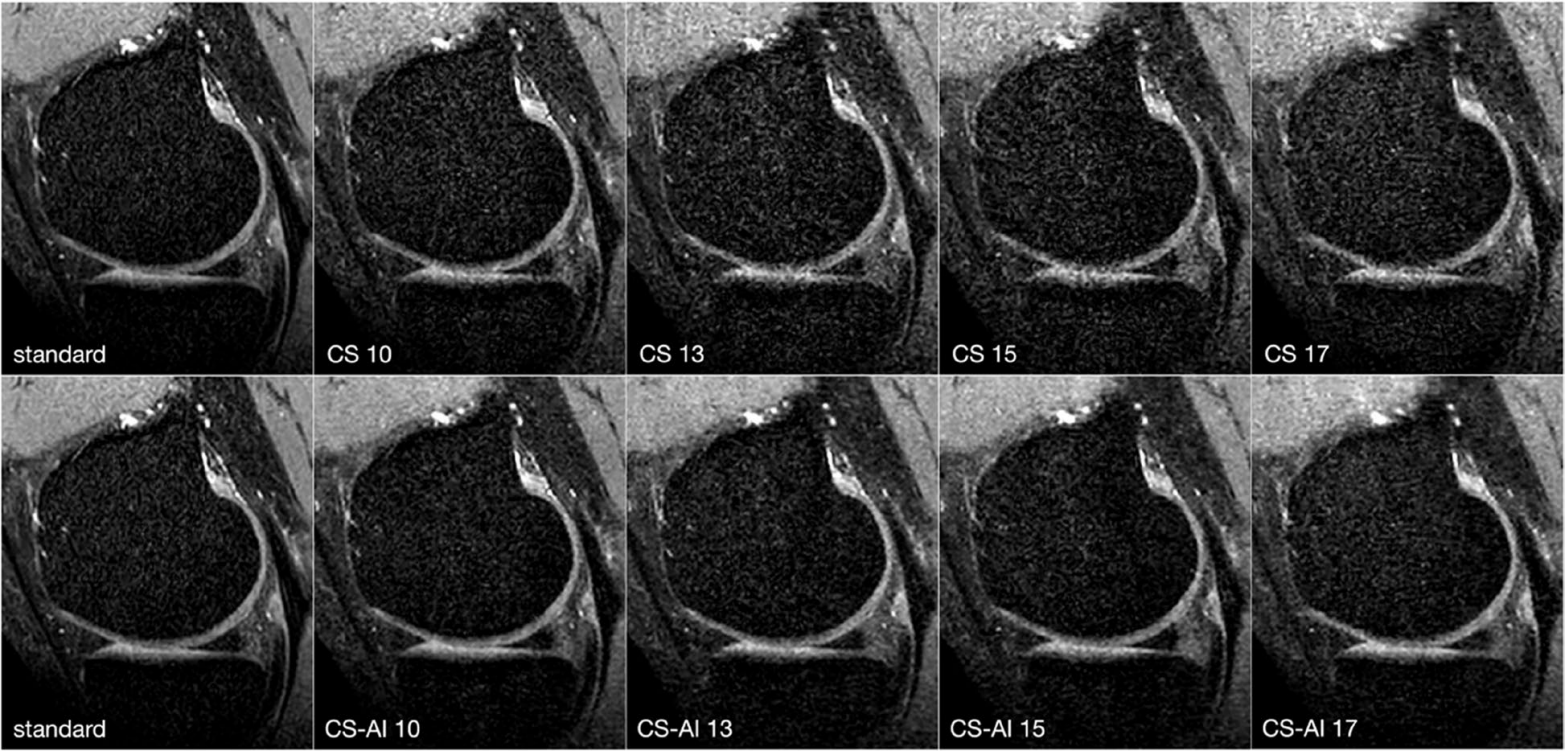
Comparison of a fat-saturated three-dimensional proton density sequence reconstructed using conventional compressed sensing (CS) and compressed sense with artificial intelligence (CS-AI) with acceleration levels of 10, 13, 15, and 17 as well as the standard sequence as reference (SENSE sequence with an acceleration factor of 2, included twice for easier comparison). Note the decreasing image quality with increasing acceleration factor. Overall, a better delineation can be observed in the lower row (CS-AI) compared to the upper row (CS)

**Fig. 2:**
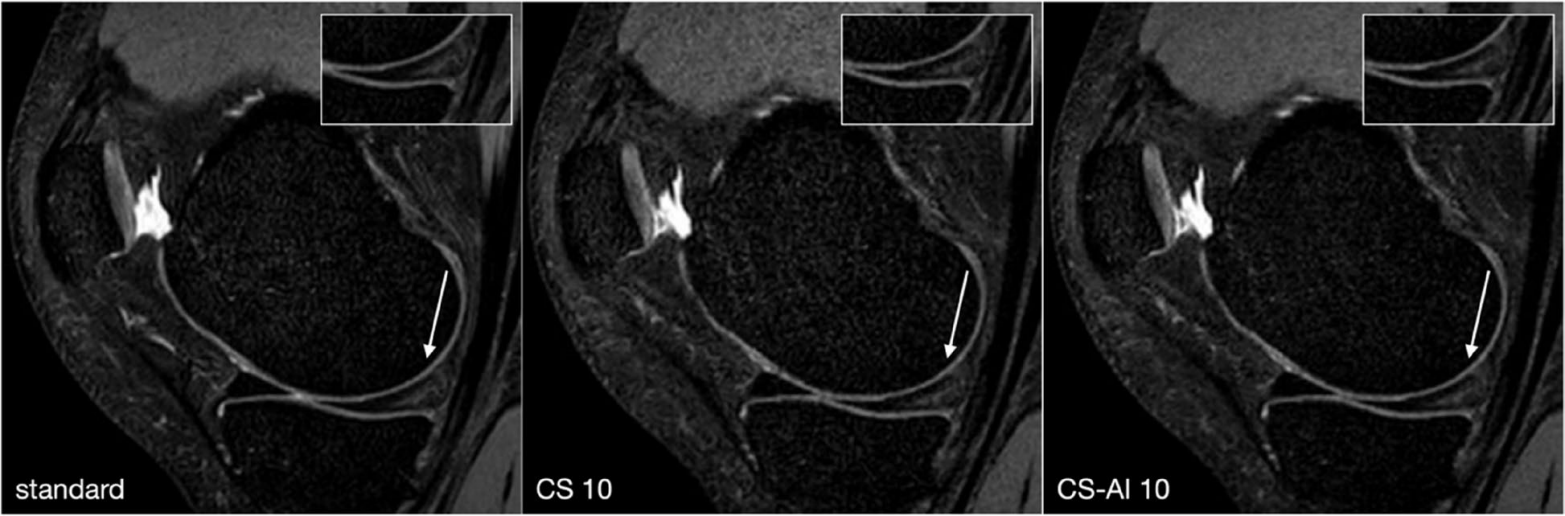
Example of a meniscal tear: linear hyperintensity in the posterior horn of the meniscus. Almost no difference can be observed between the delineation of the lesion in the reference compressed sensing (CS) SENSE sequence (standard) compared to the CS with acceleration levels of 10 (CS-10) and CS-10 with artificial intelligence (CS-AI 10)

Scan time decreased with increasing acceleration factors; for instance, from 384 s for the standard sequence to 178 s for an acceleration factor of 10 and down to 105 s for an acceleration factor of 17. An overview of the duration of the sequences is shown in Table 1. Figure 1 shows the same 3D sequence reconstructed using CS and CS-AI at the respective acceleration levels as well as the standard sense sequence as a reference. Upon review of the acquired images, it was discovered that one participant had a meniscal tear. Figure 2 shows the pathology on a 3D sequence with an acceleration factor of 10 reconstructed using CS and CS-AI compared to the reference sequence.

### Subjective image analysis

**Fig. 3:**
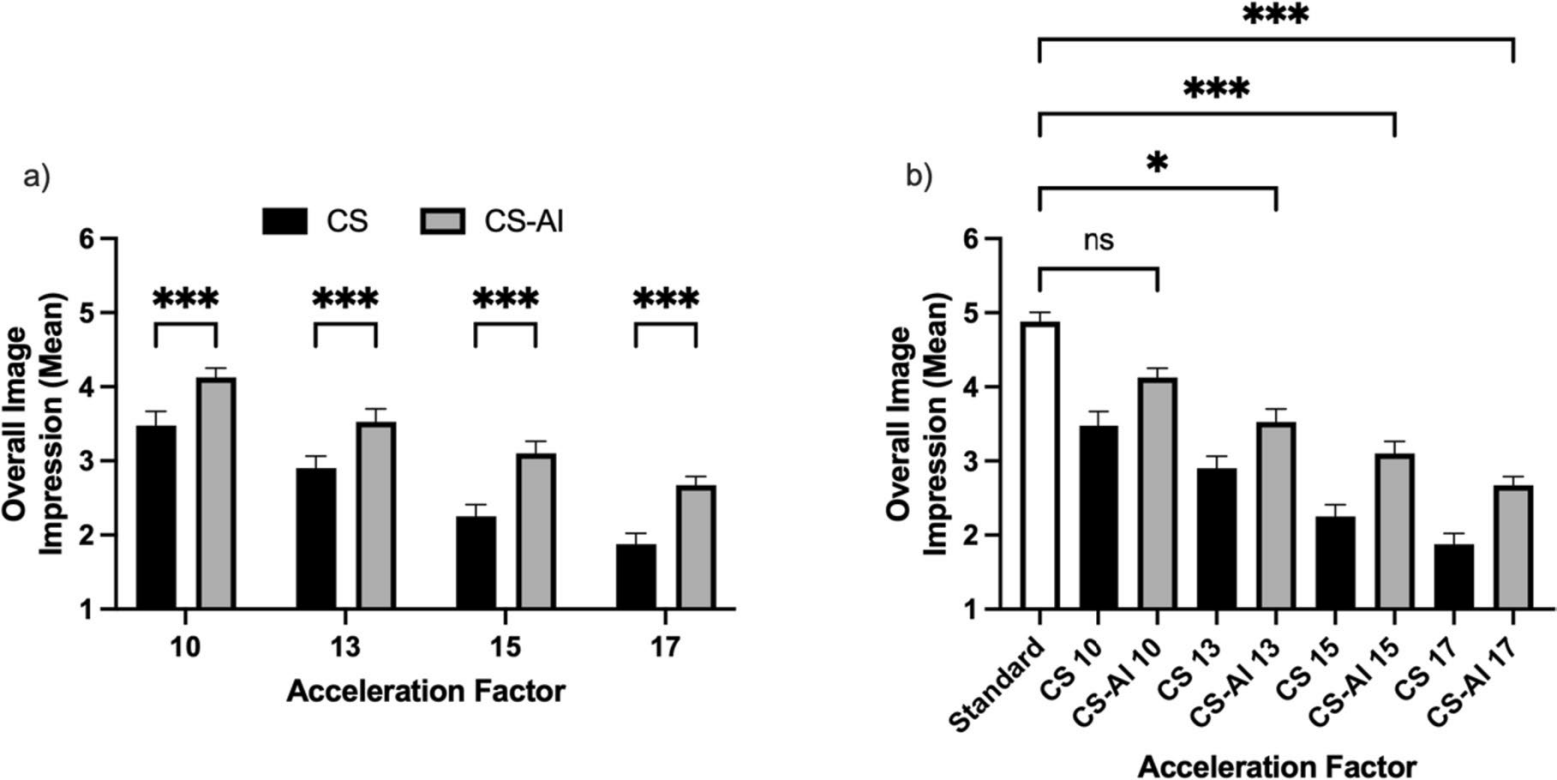
Mean subjective ratings of the overall image quality for sequences reconstructed using compressed sensing (CS) and compressed sensing combined with a deep learning-based algorithm (CS-AI). The comparison between CS and CS-AI is shown in **a**, whereas **b** shows the comparison between CS-AI and the reference sequence (SENSE sequence with an acceleration factor of 2). * *p* ≤ 0.024, ** *p* < 0.010, *** *p* < 0.001

Interrater agreement was assessed using Krippendorff´s α, indicating substantial interrater agreement for the subjective scoring for overall image impression at all acceleration factors (Krippendorff´s α = 0.71). The restricted maximum likelihood method-REML demonstrated a significant effect of the acceleration factors on the subjective measures of image quality (*p* < 0.001). Images reconstructed using CS-AI were rated significantly better than the respective sequences reconstructed using CS for all acceleration levels and all evaluated criteria (all *p* ≤ 0.001; see Table 3 and Fig. 3). Regarding the comparison to the reference sequence (CS sequence with an acceleration factor of 2), ratings for the sequences reconstructed using CS-AI did not differ significantly from the reference sequence for all evaluated criteria for an acceleration factor up to 10 (all *p* ≥ 0.999; see Table 3 and Fig. 3).

**Table 3:** Mean values and standard deviation for the subjective reading

	Standard SENSE	CS 10/ CS-AI 10	CS 13/ CS-AI 13	CS 15/ CS-AI 15	CS 17/ CS-AI 17
Cartilage					
*Standard*	4.93 ± 0.18				
*CS*		4.08 ± 0.18 − /*	3.28 ± 0.38 */*	2.90 ± 0.38 */*	2.48 ± 0.41 */*
*CS-AI*		4.48 ± 0.20 − /*	3.93 ± 0.47 */*	3.53 ± 0.41 */*	3.03 ± 0.26 */*
ACL					
*Standard*	4.90 ± 0.18				
*CS*		3.50 ± 0.34 */*	3.00 ± 0.36 */*	2.20 ± 0.50 */*	1.80 ± 0.38 */*
*CS-AI*		4.10 ± 0.34 − /*	3.50 ± 0.50 */*	2.90 ± 0.35 */*	2.70 ± 0.37 */*
PCL					
*Standard*	4.90 ± 0.29				
*CS*		3.30 ± 0.37 */*	2.70 ± 0.44 */*	2.00 ± 0.49 */*	1.50 ± 0.43 */*
*CS-AI*		3.90 ± 0.34 − /*	3.30 ± 0.44 */*	2.80 ± 0.47 */*	2.40 ± 0.43 */*
Menisci					
*Standard*	4.90 ± 0.33				
*CS*		4.00 ± 0.30 − /*	3.20 ± 0.41 */*	2.90 ± 0.45 */*	2.40 ± 0.45 */*
*CS-AI*		4.40 ± 0.29 − /*	3.90 ± 0.35 */*	3.50 ± 0.34 */*	3.00 ± 0.30 */*
Bone					
*Standard*	4.70 ± 0.41				
*CS*		3.60 ± 0.46 − /*	2.80 ± 0.41 */*	2.10 ± 0.34 */*	1.80 ± 0.41 */*
*CS-AI*		4.20 ± 0.33 − /*	3.40 ± 0.34 */*	2.80 ± 0.30 */*	2.50 ± 0.30 */*
Artifacts					
*Standard*	4.60 ± 0.50				
*CS*		3.30 ± 0.34 − /*	2.60 ± 0.41 */*	2.00 ± 0.20 */*	1.80 ± 0.30 */*
*CS-AI*		3.80 ± 0.38 − /*	3.00 ± 0.46 */*	2.60 ± 0.39 */*	2.20 ± 0.44 */*
*Overall image impression*					
*Standard*	4.90 ± 0.28				
*CS*		3.50 ± 0.41 */*	2.90 ± 0.35 */*	2.30 ± 0.34 */*	1.90 ± 0.32 */*
*CS-AI*		4.10 ± 0.28 − /*	3.50 ± 0.38 */*	3.10 ± 0.35 */*	2.70 ± 0.24 */*

*ACL* Anterior cruciate ligament, *PCL* Posterior cruciate ligament, *CS* Compressed sensing, *CS-AI* Compressed sensing combined with a deep learning-based algorithm

^*^/^*^denotes statistically significant differences (*p* ≤ 0.034) compared to the standard sequence (*before diagonal slash) or the corresponding reconstruction with the same acceleration level (CS *versus* CS-AI) (*after diagonal slash)

## Discussion

The aim of the current study was to assess the subjective image quality of conventional CS and CS with AI at different acceleration factors for 3D diagnostic MRI of the knee. The purpose was to investigate the feasibility of using CS with AI to further decrease acquisition times of knee MRI while maintaining diagnostic image quality.

The findings demonstrate that images reconstructed using CS-AI consistently achieved significantly higher ratings for subjective measurements of image quality across all acceleration levels, compared to the corresponding images reconstructed using CS. These results align with previous research employing the same algorithm. Notably, Fervers et al. observed significantly improved subjective image quality for 3D T2-weighted images of the lumbar spine when employing CS-AI, as opposed to CS-based reconstruction [20]. Moreover, studies focusing on MRI of the ankle and prostate have reported enhanced objective and subjective image quality for sequences reconstructed with CS-AI [21, 22]. While CS-AI yields images with superior subjective image quality in comparison to CS, it is important to consider that excessively high acceleration levels may compromise diagnostic quality. Thus, our study aimed to assess the images generated by CS-AI across various acceleration levels and compare them to the reference sequence currently employed in clinical practice (a sense sequence with an acceleration factor of 2). Our objective was to determine an optimal acceleration factor that produces images with subjective image quality that is not significantly different from the existing clinical standard.

Based on the subjective image quality, a 3D sequence reconstructed using CS-AI with a tenfold acceleration did not perform significantly worse than the same sequence reconstructed using sense with an acceleration factor of 2. It is important to note that the other acceleration factors (13, 15, 17) did demonstrate a significant decline in image quality compared to the standard acquisition sequence. This decline was evident to a degree where these higher acceleration levels are not currently suitable to replace the standard sequence in clinical practice. This finding underscores the trade-off between higher acceleration and image quality, which is a critical consideration for the practical application of DL-based algorithms in MRI.

Replacing the standard sensitivity encoding sequence − SENSE 2 with the CS-AI 10 sequence (384 s *versus* 178 s) would result in 54% faster image acquisition for 3D sequences. Faster image acquisition holds the potential to enhance operational efficiency within imaging centers while enhancing patient accessibility to imaging services. Moreover, reducing the duration of patient scans can effectively minimize motion artifacts, leading to improved image quality, and therefore enhanced diagnostic accuracy. Moreover, short acquisition times can increase patient comfort during MRI, possibly making it more accessible to a broader range of patients. For instance, patients suffering from claustrophobia may find MRI more tolerable with reduced scan durations.

Nevertheless, it is essential to acknowledge the limitations of our study.

First, our sample size was relatively small, consisting solely of healthy volunteers. While the results from the two readers indicate comparable delineation of anatomical structures to the reference sequences, future investigations should involve scanning patients with prevalent knee pathologies to verify the preservation of image quality for pathological findings. An ongoing concern regarding DL-based reconstruction algorithms is the potential loss of information, where abnormal anatomical findings may be substituted with normal anatomy learned from the training data. To counteract this, the multi-scale network used in this study includes the integration of a data consistency term per coil element comparing the reconstructed data with the originally acquired data to ensure consistency [17]. Studies assessing other anatomical regions, including patients with pathologies and using the same CS-AI algorithm as in our study found no evidence for loss of information. For instance, Bischof et al. found no difference in Prostate Imaging Reporting and Data System − PI-RADS scores between images reconstructed using CS-AI compared to images reconstructed using CS [22]. Nevertheless, future studies should include more participants as well as patients with different pathologies to further evaluate the accuracy of algorithm.

Second, our study only focused on a 3D proton density sequence of the knee. Whereas other studies have shown similar performance of DL-based reconstruction algorithms across different MRI sequences [23], there are also studies showing that performance can differ between MRI sequences [24]. Thus, future studies should also include other sequences besides proton density sequences (*e.g.,* T1-weighted sequences) to ensure that image quality are equally well preserved.

Third, it is important to note that our study did not incorporate objective measures of image quality. Previous research by Foreman et al. has discussed that traditional objective quality measures, such as signal-to-noise ratio, may not accurately reflect the quality of accelerated images generated through DL techniques [21]. Interestingly, Foreman et al. [21] observed an increase in signal-to-noise ratio with higher acceleration levels, despite a decrease in subjective image quality. This discrepancy could be attributed to the inherent noise reduction capabilities of DL-based reconstructions, rendering them less noisy compared to conventional reconstructions. Therefore, in our study, we relied solely on subjective image quality as the determining criterion to establish an appropriate acceleration factor that achieves comparable image quality to the reference sequence. Future investigations should explore alternative objective image quality measures that are not influenced by smoothing or denoising effects introduced by DL algorithms.

In sum, the results of our study show that the combination of DL and CS hold the potential for further scan time reduction in 3D imaging of the knee while providing overall better subjective image quality compared to CS alone. The implementation of this algorithm can help increase patient access to imaging and reduce motion artifacts by decreasing the overall time patients spend in the scanner. The results encourage further clinical investigation, extending the use cases to a clinical population and a wider range of MRI sequences. However, this study is limited by its small sample size and inclusion of only healthy volunteers, indicating the need for further research with a more diverse and larger sample.

## Data Availability

The data underlying this article cannot be shared publicly for the privacy of individuals that participated in the study. The data will be shared on reasonable request to the corresponding author.

## Abbreviations

3D: Three-dimensional
AI: Artificial intelligence
CS: Compressed sensing
DL: Deep learning
MRI: Magnetic resonance imaging
